# Imidocarb Dipropionate Lacks Efficacy against *Theileria haneyi* and Fails to Consistently Clear *Theileria equi* in Horses Co-Infected with *T. haneyi*

**DOI:** 10.3390/pathogens9121035

**Published:** 2020-12-10

**Authors:** Kelly Sears, Donald Knowles, Kelcey Dinkel, Philip W. Mshelia, Cynthia Onzere, Marta Silva, Lindsay Fry

**Affiliations:** 1 Department of Veterinary Microbiology and Pathology, Program in Vector-borne Diseases, College of Veterinary Medicine, Washington State University, Pullman, WA 99164, USA; kellyp.sears@wsu.edu (K.S.); dknowles@wsu.edu (D.K.); kelcd23@hotmail.com (K.D.); miduko@gmail.com (P.W.M.); cynthia.onzere@wsu.edu (C.O.); marta_gomeis@hotmail.com (M.S.); 2 Department of Veterinary Medicine, Ahmadu Bello University, Zaria 810253, Nigeria; 3 CureVac AG, 72076 Tubingen, Germany; 4 Animal Disease Research Unit, USDA—ARS, Pullman, WA 99164, USA

**Keywords:** equine theileriosis, *Theileria haneyi*, treatment, imidocarb diproprionate

## Abstract

Control of *Theileria equi*, the primary cause of equine theileriosis, is largely reliant on acaracide use and chemosterilization with imidocarb dipropionate (ID). However, it is currently unknown if ID is effective against *Theileria haneyi*, the recently identified second causative agent of equine theileriosis, or if the drug maintains effectiveness against *T. equi* in the presence of *T. haneyi* co-infection. The purpose of this study was to address these questions using ID treatment of the following three groups of horses: (1) five *T. haneyi* infected horses; (2) three *T. haneyi*-*T. equi* infected horses; and (3) three *T. equi*-*T. haneyi* infected horses. Clearance was first evaluated using nPCR for each *Theileria* sp. on peripheral blood samples. ID failed to clear *T. haneyi* in all three groups of horses, and failed to clear *T. equi* in two of three horses in group two. For definitive confirmation of infection status, horses in groups two and three underwent splenectomy post-treatment. The *T. equi*-nPCR-positive horses in group two developed severe clinical signs and were euthanized. Remaining horses exhibited moderate signs consistent with *T. haneyi*. Our results demonstrate that ID therapy lacks efficacy against *T. haneyi*, and *T. haneyi*-*T. equi* co-infection may interfere with ID clearance of *T. equi*.

## 1. Introduction

The apicomplexan hemoparasite *Theileria equi* is the primary causative agent of equine theileriosis and is endemic in both tropical and subtropical regions of the world. The distribution of infection is dependent on the presence of competent ticks from the genera *Hyalomma*, *Dermacentor*, *Rhipicephalus* and/or practices leading to iatrogenic transmission [1,2]. The few remaining, non-endemic countries employ stringent importation testing and prohibit entry of positive equids, resulting in “ongoing economic and regulatory challenges regarding international horse transport” [1]. This testing barrier imposes a significant economic impact on the equine industry of endemic countries that are subsequently unable to export to non-endemic countries [3].

Regardless of location during natural infection of naïve equids, *T. equi* induces clinical signs ranging from acute, severe hemolytic anemia and resultant weakness, hemoglobinuria, and death, to inapparent infection, with the natural determinants of disease severity largely unknown [4]. In an experimental setting, however, *T. equi* is invariably fatal in splenectomized equids, which develops acute, severe disease with rapidly escalating parasitemia and subsequent anemia as indicated by decline in packed cell volume (PCV)/hematocrit (HCT). These equids eventually succumb to disease due to massive hemolytic crisis because of unchecked parasite replication and subsequent erythrolysis [5,6,7]. Fortunately, horses are rarely splenectomized outside of the research setting, where splenectomy is employed as a means to verify chemotherapeutic parasite clearance or for development of parasite stabilates for future inoculations [5,6,8,9]. While *T. equi* infection of splenectomized horses is fatal, spleen-intact horses that survive acute infection transition to a persistent, asymptomatic infection state and remain silent reservoirs for transmission [4].

Immune responses that give rise to the transition from acute, severe disease to inapparent infection are only partially defined for *T. equi.* The development of specific immunoglobulin isotypes (IgG 1, 4, 7) correlates with control of parasitemia during the acute phase of infection, and additional isotypes, IgG3 and IgG5 (IgG(T)), appear after the resolution of parasitemia [10,11]. Since robust antibody responses play a role in parasite control, the primary diagnostic assay used for both importation testing and identification of carrier equids is a competitive ELISA, which measures *T. equi*-specific antibodies to EMA-1 in horse serum [8,12].

Despite the apparent role of adaptive immunity in parasite control, a successful vaccine has yet to be developed, due in large part to the relative paucity of data on the equine immune response. The significant genetic diversity within *T. equi* clades further complicates vaccine development efforts. Five distinct *T. equi* clades are currently recognized [13], and recent work has provided sufficient evidence to define a second species of equine-infective *Theileria*, known as *Theileria haneyi* [14]. In contrast to *T. equi*, *T. haneyi* (Eagle Pass strain) induces minimal clinical disease in spleen-intact horses [14,15], characterized by mild changes in PCV and occasional development of fever during the acute phase [14,15].

Epidemiological data documenting the prevalence of this new species is limited. However, it has been identified in South Africa, Nigeria, The Gambia, and in horses along the US-Mexico border [14,16,17,18]. Prevalence surveys in The Gambia, Nigeria, and South Africa have also reported co-infection of horses with *T. equi* and *T. haneyi* or triple infection with *T. equi*, *T. haneyi*, and *B. caballi* [16,17,18]. Both of these *Theileria* species cause persistent, asymptomatic infection, and both are capable of superinfection in the presence of humoral immunity to the other [15]. Long-term studies suggest that the majority of co-infected horses are unable to spontaneously clear either species, and, as there is not yet a vaccine, control strategies are centered on acaricide use, rigorous surveillance testing, and the use of chemotherapeutic drugs to clear infection [4,15].

Currently, the only medication known to be effective in achieving *T. equi* chemosterilization in vivo is imidocarb dipropionate (ID), an aromatic diamidine and carbanilide derivative [8,19,20]. Frerichs et al., initially validated the current ID treatment regimen of 4 mg/kg, administered every 72 h for 4 doses [19]. However, clearance was not achieved in all horses, even after two courses of ID [19]. Imidocarb dipropionate is also hepato- and nephrotoxic at high doses, and has significant side effects at therapeutic doses attributed to its anticholinesterase effects, including colic, diarrhea, and salivation [19,21,22]. These side effects are often ameliorated using co-administration of anti-cholinergic and anti-spasmodic medications [19,22,23]. In previous studies, equine piroplasmosis, chemosterilization, or clearance is defined by inability to detect organisms using nested PCR (nPCR) assays and/or failure to transmit the organism to a naïve, splenectomized horse via whole blood transfer [8,24]. However, neither of the aforementioned criteria are utilized by the United States or by the OIE as definitive tests for importation [4,24].

Although ID is quite effective, rare horses remain *T. equi*-positive despite two rounds of treatment, with only 1/14 horses with a Florida, USA isolate of *T. equi* failing to clear and 1/6 remaining infected with a Peruvian strain [19,20]. In a recent study of horses infected with a *T. equi* strain from Texas, USA, 24/25 horses were successfully cured after a single course of treatment, and one horse was not cleared after two rounds of ID treatment [8]. Furthermore, in vitro studies suggest the development of ID-resistance by some *T. equi* strains following ID exposure [25]. Given the recent recognition of *T. haneyi* in the U.S., the lack of information regarding its susceptibility to ID, and the presence of horses super-infected with *T. equi* and *T. haneyi* at the U.S.-Mexico border, the goals of this study were: (1) to assess whether ID treatment is an effective chemosterilization agent for *T. haneyi* infected horses and (2) to determine if *T. haneyi* co-infection interferes with ID clearance of *T. equi* infection in horses.

## 2. Results

### 2.1. T. haneyi Experimental Infection

For horses in group 1, *T. haneyi* infection resulted in mild to inapparent disease. Three of five horses developed fevers for 1–5 days post-inoculation, and a single horse developed mild decline in HCT. Clinical and erythrocyte parameters were within normal limits in all group 1 horses by day 60 post-inoculation. Horses in groups 2 and 3 exhibited similar clinical signs following stabilate inoculation of naïve horses for group 2 and *T. equi* infected horses for group 3 as previously described [15].

### 2.2. Imidocarb Diproprionate Treatment

During both courses of ID treatment, horses overall exhibited minimal negative side effects immediately following ID administration. Two group 1 horses did develop signs of colic necessitating administration of flunixin meglumine during their first round of ID treatment. Colic signs resolved with medication administration. All horses developed changes in muscle and/or liver enzymes (creatine kinase (CK), aspartate aminotransferase (AST), and gamma-glutamyl transferase (GGT)) and/or developed injection site swelling within 3–6 days of treatment (Table 1). Muscle and liver enzyme levels declined to normal prior to administration of the second course of treatment for all 3 groups, and again prior to splenectomy for groups 2 and 3. All 3 groups of horses following the second course of ID administration were asymptomatic for equine piroplasmosis and negative by blood smear cytology regardless of persistent low-level infections detected via nPCR. Groups 2 and 3 also remained asymptomatically infected at the time of splenectomy (Table 2).

### 2.3. Imidocarb Dipropionate Fails to Clear T. haneyi Infection

Following two courses of ID treatment, all horses in all three groups remained positive for *T. haneyi* by nPCR (Table 2). In a single horse from each group (3 of 11 total horses), parasitemia transiently dropped below detectable levels at 1–2 time points after the first course of treatment. However, parasitemia likely subsequently increased to sufficiently allow detection by nPCR at all successive time points following the second course of treatment and prior to splenectomy for groups 2 and 3 (Table 2).

### 2.4. Imidocarb Dipropionate Fails to Clear T. equi in a Subset of Horses Co-Infected with T. equi and T. haneyi

**Figure 1:**
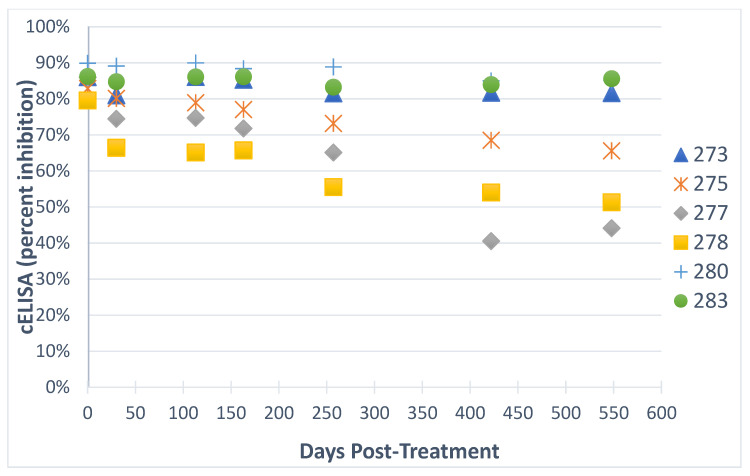
Change in *T. equi* cELISA values following two courses of imidocarb dipropionate treatment. Day zero is the day of the last dose of the second round of treatment. A horse is considered to be positive by c*ELISA* when the percent inhibition is greater than 40%.

Following two ID treatment courses, co-infected horses in groups 2 and 3 were regularly evaluated using *T. equi* nPCR and cELISA for evidence of parasite clearance. After the first round of ID therapy, 4 out of 6 horses in these two groups were negative for *T. equi* by nPCR, and 5/6 horses were negative for *T. equi* by nPCR prior to and after the second course of therapy. The *T. equi* nPCR was performed on DNA from blood collected at multiple, post-ID treatment/pre-splenectomy time points. At these time points, two horses from group 2 had variable *T. equi* nPCR results, and all three horses in group 2 maintained *T. equi* cELISA values greater than 80% (Table 2) (Figure 1). In contrast, all horses in group 3 exhibited declining values on the *T. equi* cELISA test over time prior to splenectomy (Figure 1).

To definitively determine whether ID had successfully cleared the *T. equi*-*T. haneyi* co-infected horses of *T. equi*, all six horses in groups 2 and 3 were splenectomized approximately 657–743 days after completion of the second ID treatment regimen. Intraerythrocytic *Theileria* sp. parasites were detected in all horses within seven days following splenectomy by blood smear. Horses 273 and 283 developed severe, acute disease characterized by fever, hemoglobinuria, icterus, leukocytosis (neutrophilia), thrombocytopenia, and a progressive, rapid decline in hematocrit (19–29%) that corresponded with rapidly rising parasitemia of 25–41%. Both horses were found to be positive for *T. equi* by nPCR, and were humanely euthanized.

**Figure 2:**
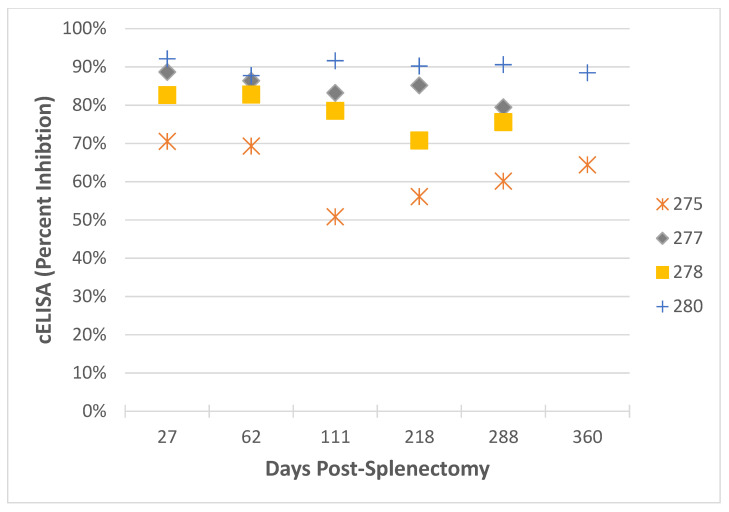
Changes in *T. equi* cELISA post-splenectomy in surviving horses. Day zero is the day of splenectomy for each horse. Splenectomies were performed asynchronously.

The surviving four horses were confirmed via nPCR to be infected with *T. haneyi* alone. Each of these animals developed moderate clinical signs consistent with equine theileriosis, including fever, icterus, variable leukocytosis (neutrophilia and monocytosis), thrombocytopenia, and anemia. Peak parasitemia was observed between days 14–19 and ranged from 7.5–14.4%. In these horses, the hematocrit declined to a nadir of 10–23.6% from days 22 to 31 post-splenectomy (Table 3). Following splenectomy, two of the four surviving horses (277 & 278) showed a marked increase in their *T. equi* cELISA values, and all surviving horses continued to have values above the positive cutoff of 40% inhibition (Figure 2). All clinical abnormalities resolved following the initial recrudescence phase post-splenectomy, and horses remained persistently, asymptomatically infected with *T. haneyi*, but not *T. equi*, 3–6 months later.

## 3. Discussion

The data presented demonstrate that imidocarb dipropionate is not effective for chemosterilization of horses infected with *T. haneyi.* More importantly, our data demonstrate that ID fails to clear *T. equi* in a subset of horses infected with both *T. equi* and *T. haneyi.* This is troubling due to the fact that both *T. haneyi* and *T. equi* are endemic in countries across the world, and several naturally co-infected horses have already been identified (unpublished data). Inter-country movement of *T. equi*-infected horses is tightly constrained, and ID therapy is a cornerstone of *T. equi* clearance for transport and trade. If ID fails to clear *T. equi* in co-infected animals, the global equine industry could face tremendous economic losses. Furthermore, persistent piroplasmosis can lead to reduced performance in horses used for athletic events or farming/traction purposes, even in the absence of other appreciable clinical signs [4,26]. The absence of any effective treatment for *T. haneyi*, and the lack of a consistently effective treatment for *T. equi* in co-infected horses, will significantly impact producers, farmers, and the international movement of equine athletes.

In our study, administration of imidocarb dipropionate resulted in local (injection site swelling) and systemic changes (muscle and liver enzyme elevations) consistent with previous studies [21,22]. These changes are associated with the hepatoxic and nephrotoxic effects of ID. Fortunately, the elevation of biochemical parameters resolved without intervention, suggesting irreversible damage was not sustained. The anticholinesterase effects (salivation, diarrhea, colic) were mitigated with Buscopan administration in our cohort, and mild colic was observed in only two horses following administration of ID [21].

The *T. equi* cELISA and nPCR for *T. equi* and *T. haneyi* were utilized prior to splenectomy to assess infection status since horses in all experimental groups remained largely asymptomatic. In general, horses that have been successfully treated for Texas-isolates of *T. equi* exhibit a decline in cELISA values over time [20,24]. However, even when infection is eliminated, it can take years for the cELISA values to fall below the 40% positive cut-off [24]. Furthermore, horses may become transiently negative by nPCR post-treatment, but subsequently test positive again after several months [27]. Thus, to confirm whether parasite clearance had occurred, we splenectomized all horses in our *T. equi*/*T. haneyi* co-infection groups several months after their second ID treatment course. Removal of splenic control allows rapid amplification of *T. equi* parasitemia, resulting in patent clinical disease in persistently infected, previously asymptomatic, horses [5,6,9,28].

ID appears to be inconsistently effective in clearing *T. equi* in the presence of *T. haneyi* co-infection. The mechanism responsible for therapeutic failure in a subset of horses is not yet known. However, authors have suggested within-host competition between resistant and non-resistant strains can play a role in the evolution of resistance [29]. Furthermore, Hansen and colleagues demonstrated that “drug sensitive strains can competitively exclude drug-resistant strains in untreated hosts” [30], and chemotherapeutic treatment can result in competitive facilitation or release of drug resistant organisms [30]. The same *T. equi* stabilate was utilized to infect all six of the superinfected horses in this study. The stabilate was developed from a splenectomized horse that had received a blood transfusion from a persistently infected horse. The ID-susceptibility of the parasites within the splenectomized horse, and the blood that infected that horse, is unknown. However, the source animal was from the group of 25 naturally infected horses in TX, of which, 24 were successfully treated with ID. Interestingly, three of four surviving horses were first infected with *T. equi* and then superinfected with *T. haneyi*, while both horses that remained dually infected were first infected with *T. haneyi* and then superinfected with *T. equi*, suggesting that order of infection could play a role in the capacity of ID to clear *T. equi.* Other research scientists have speculated on the role infection sequence may play in disease dynamics and severity [31]. However, further studies, utilizing a greater number of animals, are required to substantiate or refute this observation.

Overall, identification of effective control strategies capable of successfully eliminating vector-borne equine pathogens are growing in demand as vector species once constrained to certain regions of the world expand into previously uninhabitable territories due to climate change and environmental alteration by human and animal movement. As the development of an equine piroplasmosis vaccine remains elusive for the near future, and imidocarb diproprionate appears to be ineffective against *T. haneyi* and variably effective against *T. equi* in the presence of *T. haneyi* co-infection, additional studies are warranted to find more effective chemotherapeutic drugs capable of clearing all causative agents of equine theileriosis. Numerous compounds have been screened for in vitro efficacy against *T*. *equi*, but none have yet been assessed in clinical trials [32,33,34]. In addition, numerous new compounds have shown in vitro efficacy against related protozoa, including *Cryptosporidia* sp., *Toxoplasmosis* sp., and *Babesia* sp. [35,36]. Thus, future work should focus on expanded in vitro screening of compound libraries, coupled with in vivo equine studies to assess clearance of *T. equi* and *T. haneyi* in the equine host.

## 4. Materials and Methods

### 4.1. Horses

Eleven ponies (5 geldings and 6 mares), were used in this study, and all originated from a close research herds at either the University of Idaho or Washington State University. Horses resided on premises that were tick-free and were maintained on double-fenced pastures prior to being stalled for the research study. Horses were divided into three groups. Age at initial inoculation ranged from yearlings to 4.5 years old. All horses were confirmed *T. equi* and *T. haneyi*-negative using nPCR on whole blood (described below) prior to the onset of the study. Group assignments by horse number are shown in Table 4. Horses in group 1 were infected with *T. haneyi* only. Within this group, horse 270 was infected with *T. haneyi* (*Eagle Pass* isolate) via whole blood transfusion from a known positive horse [14], and the remaining four horses were infected with *T. haneyi* (*Eagle Pass*) via intravenous injection of 2–4 mL of stabilate derived from the blood of a known positive horse (8.8% percent parasitized erythrocytes (PPE) [14]. Horses in groups 2 and 3 were infected via intravenous inoculation with 2 mL *T. haneyi* (*Eagle Pass*) infected frozen erythrocyte stabilate, (12% PPE) and 1 mL *T. equi* (*Texas* isolate) stabilate (38% PPE). Group 2 was infected with *T. haneyi* first, and then superinfected with *T. equi*, and group 3 was initially infected with *T. equi* and then superinfected with *T. haneyi* [15]. For all stabilate infections, stabilates were thawed slowly at room temperature, combined with either 10% normal horse serum in phosphate buffered saline or autologous serum and then injected intravenously over 2–3 min. For all horses, *T. haneyi* and *T. equi* infection status was confirmed using nPCR at multiple time points prior to treatment, and all horses were 3–7 years old at the time of treatment. Horses were housed in pairs. All animal experiments were approved by the Washington State University and University of Idaho Institutional Animal Care and Use Committees, ASAF numbers 4973 and 6241 (WSU) and 2016–18 and 2016–28 (UI). Ethics Approval: All animal experiments were approved by the Washington State University and University of Idaho Institutional Animal Care and Use Committees, ASAF numbers 4973 and 6241 (Washington State University) and 2016–18 and 2016–28 (University of Idaho).

### 4.2. Blood Collection

Blood was collected via jugular venipuncture immediately prior to inoculation and serially following inoculation. Blood was also collected immediately prior to each imidocarb dipropionate treatment and at monthly intervals thereafter. Blood was collected into both ethylenediaminetetraacetic acid (EDTA) and serum separator tubes and processed for use in diagnostic assays within two hours of collection. Serum separator tubes were centrifuged at 1500× *g* for 10 min following clot formation. Serum and whole blood samples were subsequently divided into aliquots for complete blood counts (CBC), serum chemistry panels, blood smears, serologic assays, and DNA isolation for PCR. Serum and DNA samples were stored at −20 °C.

### 4.3. Imidocarb Dipropionate Treatment

Horses underwent initial ID treatment (four doses, each 72 h apart) 6 to 56 months post-inoculation. Approximately 10–15 min before ID injection, horses were pre-treated with 0.3 mg/kg N-butylscopolammonium bromide (Buscopan^®^, Boehringer Ingelheim, Duluth, GA, USA) via intramuscular (IM) injection in the neck. Imidocarb dipropionate (Imizol—Merck Animal Health (Kenilworth, NJ, USA) & Imochem120—Interchemie (Venray, The Netherlands) [8,37] was then administered IM (4 mg/kg) on the opposite side of the neck. All horses were closely monitored for one hour after ID administration for any adverse effects (colic, diarrhea, dyspnea, ptyalism, changes in attitude), and then subsequently monitored daily for injection site reactions and every 7–14 days via serum chemistry panel for evidence of renal or hepatic toxicity until values returned to within the normal range. All horses received a second course of ID treatment (4 mg/kg IM q 72 h for 4 doses) 2–4 months after completion of the initial treatment course. Horses that developed colic post-ID administration were treated with flunixin meglumine 1.1 mg/kg IV (Prevail™ Bimeda-MTC Animal Health, Inc.; Cambridge, ON, Canada).

### 4.4. T. haneyi Nested PCR (Genomic DNA)

DNA was isolated from whole blood using the DNeasy Blood and Tissue Kit (Qiagen, Inc., Venlo, The Netherlands) per the manufacturer’s instructions, and was stored at −20 °C until analysis by nPCR. The nPCR was designed to amplify a gene target absent from the *T. equi* genome and was performed as previously described [14,15]. Primers utilized for the reaction were the following: External Forward 5′ CCATACAACCCACTAGAG 3′, External Reverse 5′ CTGTCATTTGGGTTTGATAG 3′, Internal Forward 5′ GACAACAGAGAGGTGATT 3′, and Internal Reverse 5′ CGTTGAATGTAATGGGAAC 3′ [15]. Briefly, for the external reaction, 12.5 µL of 2X DreamTaq PCR Master Mix (Thermo Scientific, Waltham, MA, USA), 1 µL (10 µM) Fwd external primer, 1 µL (10 µM) Rev external primer, 5.5 µL of nuclease-free water, and 5.0 µL of DNA were combined. The external reaction was carried out under the following conditions: 95 °C for 4 min, then 35 cycles of 95 °C for 20 s, 63.5 °C for 30 s, and 72 °C for 20 s, and final extension at 72 °C for 7 min. For the internal reaction, 12.5 µL 2X DreamTaq Green PCR Master Mix (Thermo Scientific, Waltham, MA, USA), 1 µL (10 µM) Fwd internal primer, 1 µL (10 µM) Rev internal primer, 9.5 µL of nuclease-free water, and 1 µL of external reaction product were used. The internal reaction was carried out under the following conditions: 95 °C for 4 min, then 35 cycles of 95 °C for 20 s, 58.1 °C for 30 s, and 72 °C for 20 s, and final extension at 72 °C for 7 min. For all *T. haneyi* nPCR, the positive control consisted of genomic DNA isolated from a *T. haneyi* infected splenectomized horse that had been confirmed to be positive by both light microscopy and nPCR. The negative control is nuclease-free water.

### 4.5. T. equi Nested PCR (Genomic DNA)

nPCR for *T. equi* utilized primers for the *ema-1* gene, which is absent from the genome of *T. haneyi*, and was performed as previously described [14,38]. Briefly, for the external reaction, 12.5 µL of 2X DreamTaq PCR Master Mix (Thermo Scientific, Waltham, MA, USA), 1 µL 10 µM Fwd external primer, 1 µL of 10 µM Rev external primer, 5.5 µL of nuclease-free water, and 5 µL of genomic DNA were used. The external reaction was carried out under the following conditions: 95 °C for 5 min, then 25 cycles of 95 °C for 20 s, 60 °C for 20 s, 72 °C for 20 s, and final extension at 72 °C for 10 min. For the internal reaction, 12.5 µL of 2X DreamTaq Green PCR Master Mix (Thermo Scientific, Waltham, MA, USA), 1 µL 10 µM Fwd internal primer, 1 µL of 10 µM Rev internal primer, 9.5 µL of nuclease-free water, and 1 µL of external reaction product were used. The internal reaction was carried out under the following conditions: 95 °C for 5 min, then 25 cycles of 95 °C for 5 s, 60 °C for 5 s, 72 °C for 5 s, and final extension at 72 °C for 10 min. For all *T. equi* nPCR, the positive control utilized was genomic DNA isolated from *T. equi* tissue culture that is maintained by our lab. The negative control is nuclease-free water.

### 4.6. T. equi cELISA

The World Organization for Animal Health (OIE) and United States Department of Agriculture (USDA)-approved *T. equi* regulatory diagnostic test, a competitive, enzyme-linked, immunosorbent assay (cELISA), was performed using a commercially available kit (VMRD, Pullman, WA, USA) as directed by the manufacturer. A positive result is defined as >40% inhibition by the manufacturer. This assay was performed at regular intervals on serum from infected horses to monitor the serologic response to *T. equi.* Specifically, on days 30, 113, 163, 257, 422, and 548 after the second round of treatment. cELISA values were subsequently evaluated post-splenectomy on a time point within the following ranges due to surgeries occurring asynchronously: Days 27–33, 54–62, 111–153, 218–239, 288–309, and 360–381.

### 4.7. Splenectomy of ID-Treated, Co-Infected Horses—T. equi Clearance Confirmation

Following the second course of ID treatment, horses in groups two and three underwent splenectomy at the Washington State University Veterinary Teaching Hospital [39] in order to definitively determine whether ID treatment cleared *T. equi* infection. All group two and three horses were splenectomized 657–743 days post-treatment. Prior to surgery, each horse underwent a pre-operative physical exam, complete blood count, serum chemistry panel, ECG, and rebreathing exam to ensure it was fit for surgery. Post-operatively, horses received phenylbutazone (4.4 mg/kg) by mouth once daily for 5 days to minimize pain and were housed in stalls for 60 days following surgery, or until euthanasia.

### 4.8. Monitoring for T. equi and T. haneyi Recrudescence

Following surgery, horses were monitored carefully for signs of acute theileriosis as removal of the spleen enables amplification of parasitemia in asymptomatic, persistently *T. equi*-infected horses [5,7]. Monitoring consisted of twice daily physical exams, daily CBC, weekly chemistry panel, and daily blood smear to detect inappetence, tachycardia, tachypnea, fever, icterus, hematuria/hemoglobinuria, parasitemia, anemia, and hyperbilirubinemia [4]. Horses were euthanized if they developed severe clinical signs refractory to supportive care, or when parasitemia levels, based on evaluation of Giemsa-stained blood smears at 100 × magnification using oil-immersion, were greater than 25%. In this study, the percent parasitemia was determined using the following equation: ((Total parasites in 5 fields)/(Erythrocyte count in ¼ of a field × 20)) × 100.

## Figures and Tables

**Table 1:** Summary of incidence of elevation in serum enzymes and injection site reactions following each 4-dose series of ID treatment.

		1st Course of Treatment				2nd Course of Treatment			
Horse Number	Group	CK	AST	GGT	Injection Site Swelling	CK	AST	GGT	Injection Site Swelling
270	1	↑	↑		N	↑	↑		N
364	1	↑	↑		N	↑	↑	↑	N
776	1	↑	↑	↑	Y				N
777	1	↑	↑		Y				N
784	1	↑	↑		Y				N
273	2	↑			Y		↑		Y
280	2	↑			N	↑			N
283	2	↑	↑		N	↑			N
275	3	↑	↑	↑	Y	↑	↑		Y
277	3	↑	↑	↑	Y	↑	↑	↑	Y
278	3	↑	↑	↑	N	↑	↑	↑	Y

↑ = Elevation, Y = Yes, N = None.

**Table 2:** *T. haneyi* and *T. equi* nPCR results at serial time points following each course of ID treatment.

***T. haneyi* nPCR**							
**Horse No.**	**Group**	**Pre-Treatment 1**	**Post-Treatment 1 ^a^**	**Pre-Treatment 2**	**Post-Treatment 2 ^a^**	**Pre-Surgery**	**Post-Surgery**
270	1	+	+	+	+	N/A	N/A
364	1	+	+	+	+	N/A	N/A
776	1	+	+	+	+	N/A	N/A
777	1	+	+	+	+	N/A	N/A
784	1	+	+	+	+	N/A	N/A
273	2	+	+	+	+	+	+
280	2	+	+	+	+	+	+
283	2	+	+	+	+	+	+
275	3	+	+	+	+	+	+
277	3	+	+	+	+	+	+
278	3	+	+	+	+	+	+
***T. equi* nPCR**							
**Horse No.**	**Group**	**Pre-Treatment 1**	**Post-Treatment 1 ^a^**	**Pre-Treatment 2**	**Post-Treatment 2 ^a^**	**Pre-Surgery**	**Post-Surgery**
273	2	+	+	−	−	+/−	+
280	2	+	−	−	−	−	−
283	2	+	+	+	+	+/−	+
275	3	+	−	−	−	−	−
277	3	+	−	−	−	−	−
278	3	+	−	−	−	−	−

^a^ Post-treatment time points were 40–60 days following the last dose of ID for the associated course of treatment. “+” = Positive, “−” = Negative, N/A = Not Applicable.

**Table 3:** Summary of hematocrit and parasitemia post-splenectomy.

Horse Number	Group	Time to Peak PPE (Days)	Peak PPE (%)	HCT Nadir (%)	Outcome	*T. equi* nPCR
280	2	17	7.69	17.8	Survived	Negative
273	2	9	25.1	19.5	Euthanized	Positive
283	2	10	40.46	28.9	Euthanized	Positive
275	3	14	7.5	23.4	Survived	Negative
277	3	17	10.58	12.6	Survived	Negative
278	3	19	14.4	10	Survived	Negative

**Table 4:** Horses utilized in the study.

Horse ID	Group Number	Inoculation
270	1	*T. haneyi*
364		*T. haneyi*
776		*T. haneyi*
777		*T. haneyi*
784		*T. haneyi*
273	2	*T. haneyi*/*T. equi*
280		*T. haneyi*/*T. equi*
283		*T. haneyi*/*T. equi*
275	3	*T. equi*/*T. haneyi*
277		*T. equi*/*T. haneyi*
278		*T. equi*/*T. haneyi*
